# Effects of Short-Term Dynamic Balance Training on Postural Stability in School-Aged Football Players and Gymnasts

**DOI:** 10.3389/fpsyg.2021.767036

**Published:** 2021-11-17

**Authors:** Rouven Kenville, Tom Maudrich, Sophie Körner, Johannes Zimmer, Patrick Ragert

**Affiliations:** ^1^Institute for General Kinesiology and Exercise Science, Faculty of Sport Science, University of Leipzig, Leipzig, Germany; ^2^Department of Neurology, Max Planck Institute for Human Cognitive and Brain Sciences, Leipzig, Germany

**Keywords:** dynamic balance training, posturography, postural stability, football, gymnastics

## Abstract

Static and dynamic balance abilities enable simple and complex movements and are determinants of top athletic performance. Balance abilities and their proficiency differ fundamentally with respect to age, gender, type of balance intervention, and type of sport. With this study, we aim to investigate whether 4weeks of dynamic balance training (DBT) improves static balance performance in school-aged gymnasts and football players. For this purpose, young male gymnasts (*n*=21) and male football players (*n*=20) completed an initial static balance assessment consisting of two one-legged stance (left and right foot) and two two-legged stance (eyes open and eyes closed) tasks. Subsequently, all participants underwent a 4-week intervention. DBT consisting of nine individual tasks was performed two times per week. Another static balance assessment followed 1day after the last training session and retention was assessed 2weeks later. Dynamic balance scores and total path length were analyzed *via* rank-based repeated measures designs using ANOVA-type statistics. The influence of factors GROUP and TIME on the static and dynamic balance performance was examined. Prior to DBT, young gymnasts showed better static balance performance than football players. However, after intervention, both groups improved in both one-legged stance tasks and also had high retention rates in these tasks. No significant improvements were seen in either group in the two-legged balance tests. Both groups improved in the dynamic balance tasks, although no differences in learning rates were evident. Our findings imply an inter-relationship between both static and dynamic balance components. Consequently, training regimes should include both balance components to facilitate early development of balance ability.

## Highlights

– Initially, gymnasts outperformed football players in static balance.
– Dynamic balance training improved static balance in both groups.
– Learning rates did not differ between both groups.

## Introduction

Successful execution of fundamental movements, as well as sport-specific skills, depends critically on an individual’s ability to maintain or adjust balance (Faigenbaum et al., 2015). Balance comprises both static and dynamic components. Static balance is generally defined as the ability to stabilize the center of mass in relation to the base of support (Horak, 1987), whereas dynamic balance relates to stability maintenance during the performance of dynamic movements (DiStefano et al., 2009). Enhanced abilities in both domains have been associated with top athletic performance (Hrysomallis, 2011). Although athletes generally possess better balance ability, recent research suggests differences in balance ability between athletes across various disciplines. For example, combined evidence of several studies investigating balance abilities in athletes of different sports implies superior balance performance in adult gymnasts compared to football players, swimmers, and basketball players (Hrysomallis, 2011). Such differences are potentially rooted in distinct training regimes, as well as skill requirements that lead to sport-specific sensorimotor adaptations (Bressel et al., 2007). Balance ability is developed in childhood and decreases over the lifespan (Bellafiore et al., 2011). Typically, balance ability progresses in a gradual manner until early adolescence (Largo et al., 2001), given that it is closely related to the development of specific systems involved in postural control (Butz et al., 2015). Children as young as 9years of age have been shown to exhibit adult postural control strategies, suggesting an early manifestation of adult balance behavior (Shumway-Cook and Woollacott, 1985).

Nevertheless, balance training in children is a heterogeneous matter, particularly due to varying maturation processes across age groups (Granacher et al., 2011). Previous studies indicated that the success of classical balance training depends on the age of the children, assuming and showing that children under 8years of age generally did not show any improvement in their balance ability as a result of balance training (Granacher et al., 2011). On the other hand, a study by Wälchli et al. (2018) demonstrated that balance training tailored to children can indeed improve postural stability in 6–7year old children. These results indicate that the success of balance training in children depends also on motivational factors. In addition to age and motivation, the specificity of the training plays an important role. Many studies imply that balance is a task-specific and not a general ability (Giboin et al., 2015). A core study in this context is Horak’s clinical test battery (Horak et al., 2009). Here, 36 balance tests were divided into six balance categories. Results on patients showed no relationships between the categories. However, it must be noted that this concerns effects in clinical and not in healthy populations. In healthy participants, Giboin et al. (2015) had two training groups train on two different devices. The results showed that the two groups improved only in the trained tasks. Similarly, 6weeks of slackline training resulted in specific improvements in slackline performance but not improvements in non-specific balance tasks (Donath et al., 2013). A review by Kümmel et al. (2016) also concluded that balance training only achieved specific effects. On the other hand, a study by Freyler et al. (2016) compared the specificity between sensorimotor and reactive balance training. Here, it was again shown that the improvements of the two training groups were specific to the task. However, the reactive balance training group showed better adaptations to a cognitive interference task (Freyler et al., 2016). This implies a transfer effect of reactive balance training in dual-task settings. Interestingly, studies on cross-education, i.e., the transfer of training effects from the trained to the untrained limb, imply that the transfer effects of balance training may not be ruled out after all (Paillard, 2017), as some studies show positive effects of unilateral training on balance ability of the untrained side (Kim et al., 2011). Still, these results need to be verified by further studies.

Since balance develops early and its shaping is closely related to the sport and training environment, current research is focused on optimizing balance training regimens to facilitate early beneficial balance ability development in young athletes across a variety of sports. Among the common training, approaches are static and dynamic balance training (DBT) programs. Both methods have been successfully used to improve balance in healthy children and adults (Bellafiore et al., 2011; Butz et al., 2015). Many studies have examined the effects of static balance training to improve static balance and DBT to improve dynamic balance (Bressel et al., 2007). An extension of this approach is to examine the effects of both methods on the other component, i.e., the effects of DBT on static balance and vice versa, which is an important aspect in terms of uncovering potential interrelationships between the two methods. However, the question of potential transfer effects between the two main components of balance also arises in this context. Encouraging evidence demonstrates positive effects of short-term, i.e., 4–5weeks of DBT on static balance in older (Battaglia et al., 2010) and overweight participants (Bellafiore et al., 2011). In addition, transfer effects in balance training are often associated with improvements in other abilities, such as strength (Gusi et al., 2012). Dynamic balance training in particular can increase strength ability (Hamed et al., 2018) and thus potentially facilitate transfer effects from dynamic to static balance ability. Investigating transfer effects of DBT on static balance seems particularly relevant, considering that many sports involve a variety of constantly changing static and dynamic balance demands, and thus young athletes potentially benefit from variable balance training. Importantly, a recent meta-analysis concluded that improvements in static and dynamic balance performance are irrespective of training status (Gebel et al., 2018). This means that young athletes can generally benefit from balance training despite their better initial balance performance compared to untrained individuals.

Previous studies have investigated the influence of balance training in young athletes (Brachman et al., 2017). For instance, various balance training interventions have been shown to improve aspects of static and dynamic balance in young gymnasts (Dobrijević et al., 2016), football players (Cankaya et al., 2015; Heleno et al., 2016), volleyball players (Pau et al., 2012), and tennis players (Sannicandro et al., 2014). Crucially, potential transfer effects from DBT to static balance ability have not yet been studied in young athletes. With this study, two contrasting sports with moderate to high balance requirements (football and gymnastics) were investigated to identify potential transfer effects between DBT and static balance ability. First, this is justified by the different balance requirements within both sports that expand the potential range of applications of the intervention (Hay, 1978; Orchard, 2002). Furthermore, there are many studies on balance ability within football players and gymnasts in adulthood, which facilitates the derivation of hypotheses, as well as the interpretation and contextualization of potential findings. Comparing the balance abilities of both types of sports, it can be stated that gymnasts show a better static balance ability than soccer players (Paillard, 2017), whereas no results exist regarding dynamic balance ability.

Consequently, regarding the fine-tuning of such training methods in the context of early athletic development, unanswered questions remain regarding (A) the effect of dynamic balance training on static balance performance in young athletes and (B) the effect of such methods on young athletes of different sport disciplines.

Against this background, the present study aimed at uncovering the effects of a 4-week DBT program on static balance performance in school-aged (9–11years) football players and gymnasts. In relation to the outlined research (Paillard, 2017), we anticipated the initial performance of young gymnasts to be better compared to young football players. Furthermore, we expected improved static balance performance in both groups after completion of the 4-week DBT program because comparable effects have been demonstrated in both elderly (Battaglia et al., 2010) and clinical populations (Bellafiore et al., 2011). Additionally, DBT has been shown to increase other fundamental motor abilities (i.e., strength), which could aid in the facilitation of static balance performance (Hamed et al., 2018). Since adult gymnasts have been shown to have better balance compared to adult football players, we expected that learning rates in learning the dynamic balance tasks would favor the young football players. This is derived from the inverse relationship between the initial learning rates and baseline motor performance and/or ceiling-effects of motor learning (Dayan and Cohen, 2011). Finally, we expected both groups to have high retention rates given the fact that children generally demonstrate high retention abilities in balance tasks (Rodríguez-Negro et al., 2020).

## Materials and Methods

### Ethical Approval

This study was supported by the Local Ethics Committee of the University of Leipzig (ref. nr. 359/16-ek). All participants and their legal guardians gave written informed consent to participate in the experiment, according to the Declaration of Helsinki.

### Participants

Initially, we performed a sample size estimation using G*Power 3.1 (Faul et al., 2009) based on previous results of balance training in children (Wälchli et al., 2018) using the following parameters: for test family=*F*-test and statistical test=repeated measures ANOVA, a power value (probability of correctly rejecting a false null hypothesis) of 0.8 was chosen given a type I error rate of *α*=5%. Additionally, the effect size (*f*) was set to 0.27, as a previous related study reported values in this range (Wälchli et al., 2018). The estimated minimum sample size to obtain sufficient test power was *n*=24. A total of 41 male participants [age (median±interquartile range, IQR): 10.2±1.6years] were enrolled in the present study. Participants were recruited through public advertisement based on the following inclusion criteria: age 9–12years, neurological healthy, no history of injuries of the lower extremities (based on self-reports of the legal guardians). Furthermore, participants were separated into two groups according to their participation in organized sports as member of an official sports club: a football group (FG; *n*=20; age: 10.3±1.7years; height: 141.5±9.1cm; weight: 31.5±5.6kg; 5.1±2.1 training years) and a gymnastics group (GG; *n*=21; age: 10.2±1.2years, height: 138.0±7.0cm; weight: 30.0±3.0kg; 6.0±0.8 training years). All participants trained 3days each week and competed at least at the regional level. Information about the participants’ athletic background and training frequency was collected with a questionnaire prior to the experiment. The handedness of participants was assessed using the Oldfield handedness inventory (Oldfield, 1971) and expressed by the laterality quotient (LQ).

### Experimental Procedure

**Figure 1:**
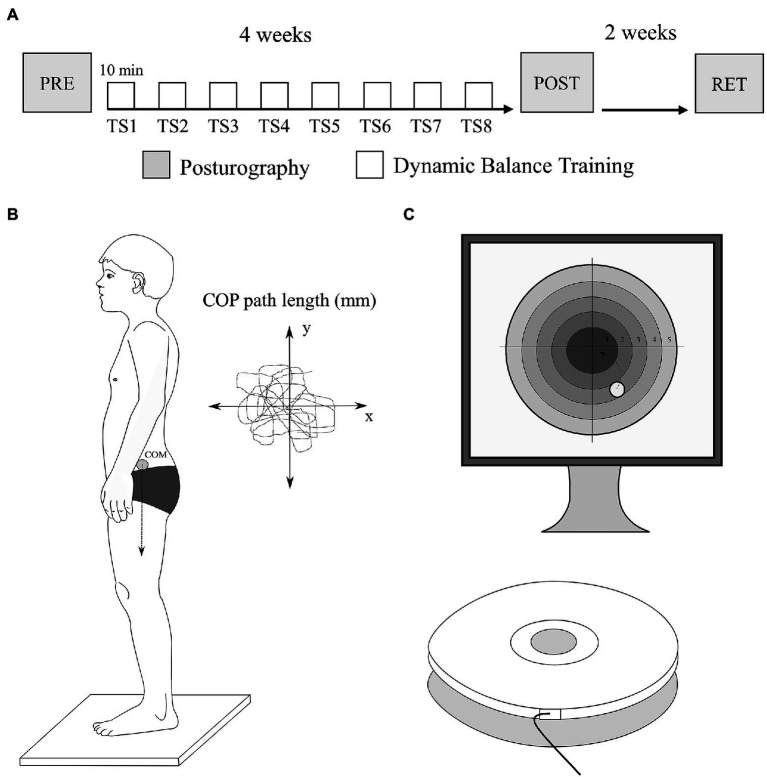
Experimental design and setup. **(A)** Dynamic balance training was performed for 4weeks over eight training sessions (TS), each lasting approximately 10min. Before and after the training period, postural stability was assessed in two-legged and one-legged stance conditions. Furthermore, 2weeks later, retention was assessed again using posturography. **(B)** Posturography was performed using the Wii Balance Board. The total center of pressure (COP) path length was recorded over a trial length of 20s as main outcome parameter of postural stability. **(C)** Dynamic balance training was performed with the challenge disk. The main goal concerning the entire dynamic balance training was to track a red circular target zone with a green point cursor (corresponding to the COP) by way of shifting the bodyweight in different directions. The cursor movement could be seen by the participants on a PC screen placed in front of them.

Participants completed a total of eight training sessions twice a week of DBT utilizing the commercially available training device Challenge Disc (CD, MFT Bodyteamwork GmbH). One training session lasted approximately 10min and was performed at comparable day times. One day before the first training session (PRE) and 1day after the last training session (POST), posturography of all participants was assessed using a Wii Balance Board (WBB, Nintendo). Additionally, retention of balance training effects on postural stability was tested after 2weeks of no balance training again using the WBB (RET; Figure 1A). All experiments were conducted in Leipzig in the period from August 2016 to October 2016.

### Posturography Assessment: Wii Balance Board

Postural stability was assessed using the WBB and the commercially available software STABLE (STAnding BaLance Evaluation – STABLE by pro-WISS, Bochum, Germany) in four different standing positions (Figure 1B). Therefore, participants stood barefoot in the middle of the WBB, shoulder-width apart and were instructed to fix their gaze on a cross attached to a wall 3m from the plate and to stand as still as possible for 20s (trial duration) in each position. A trial duration of 20s was previously shown to provide high test–retest reliability in force-plate based posturography assessments (Le Clair and Riach, 1996). Additionally, all participants were asked to keep their arms close to the body. The following four standing tasks commonly used in the literature were performed: Two-legged stance eyes open (TLS-EO), two-legged stance eyes closed (TLS-EC), one-legged stance with the right leg (OLS-R), and one-legged stance with the left leg (OLS-L) as recommended by Clark et al. (2010). Before each trial, the WBB was newly calibrated to account for the weight of each participant. The WBB functions similar to a force plate which means, that it measures ground reaction forces in three directions (*x*, *y*, and *z*) with a sampling frequency of 50Hz. Furthermore, the torque in each direction is computed to analyze deviations of the participant’s center of pressure (COP) from the center of the WBB. The total path length of COP deviation over the course of each trial (20-s duration) was used as the compound parameter for postural stability for further analysis. Previous investigations confirmed that the WBB can be used as a valid and reliable tool for assessing standing balance (Clark et al., 2010; Kalisch et al., 2011; Chang et al., 2013).

### Dynamic Balance Training: Challenge Disc

Dynamic balance training was performed twice a week for eight training sessions using the CD and its complementary training software COORDI (MFT Bodyteamwork). The CD is an established tool concerning balance performance assessment and has been used in a number of studies (Bayer et al., 2017; Seidel et al., 2017; Akbas and Mummolo, 2021). It is made up of a circular platform (440mm diameter) with an inclination sensor that is connected to a fixed base plate *via* an elastic center connection (Figure 1C). The unstable platform’s tilting must be swiftly countered by systematic counter-movements in the transverse and sagittal axis. The inclination sensor measures deviations of the COP in a range of 20 degrees with a sampling frequency of 100Hz. The initial position of the participants on the CD was a two-legged stance with slightly bent knees. The main goal concerning the entire DBT was to track a red circular target zone with a green point cursor (corresponding to the COP) by way of shifting the bodyweight in different directions. The cursor movement could be seen by the participants on a PC screen placed in front of them. DBT consisted of nine different protocols, which were performed in a fixed order. Within each protocol, the target zone moved in a different and specific way (i.e., medial-lateral, anterior–posterior, clockwise, anti-clockwise, and random).

All participants performed the balance training only in bipedal stance using COORDI level 3 throughout the whole training process, meaning that the difficulty of the DBT was not changed between training sessions. All training sessions were supervised by the same researcher. All protocols lasted 20s interspaced by 7s of rest. During each 20-s trial, the time participants correctly tracked the target zone was measured and was finally summarized for all nine training protocols as a compound measure for further analysis. One training session lasted approximately 5min, and all training sessions were performed at comparable daytimes throughout four training weeks. All participants successfully completed all eight training sessions of the DBT.

### Statistical Analyses

Due to the non-normal distribution of the majority of the variables as assessed by Shapiro-Wilk testing, non-parametric tests were used. All statistical analyses were performed in RStudio (Team, 2020).

Demographic and anthropometric variables (age, weight, height, training years, and LQ) and the initial performances in all four posturography conditions (TLS-EO, TLS-EC, OLS-R, and OLS-L) were compared pairwise between FG and GG using Mann-Whitney-U testing. The effect size was expressed by the rank biserial correlation.

Data from posturography (COP path length) of POST and RET were normalized to values obtained at PRE to investigate improvement rates of measured variables induced by DBT. Normalization was also done for all training sessions during DBT, again to capture relative improvement rates. This means that all consecutive training sessions were normalized to the training scores initially obtained at the first training session. Subsequently, the R-package “nparLD” was implemented to non-parametrically analyze the data according to a rank-based repeated measures design using ANOVA-type statistics (ATS), with the denominator degrees of freedom set to infinity (Brunner et al., 2002; Noguchi et al., 2012). This is a necessary step to improve the robustness of ATS since the use of finite denominator degrees of freedom can result in higher type I errors (Bathke et al., 2009).

Learning scores of DBT were analyzed using a non-parametric repeated measures ATS with the between-subject (whole-plot) factor GROUP (FG and GG) and the within-subject (sub-plot) factor TIME (training sessions 1–8).

Similarly, posturography results (COP path length) measured before and after DBT were investigated using non-parametric repeated measures ATS with the between-subject (whole-plot) factor GROUP (FG and GG) and the within-subject (sub-plot) factor TIME (PRE, POST, and RET).

The effect size *A*, a measure of stochastic superiority was computed for pairwise *post-hoc* comparisons of the ATS (Vargha and Delaney, 2000). The interpretation benchmarks of *A* are small effect ~0.56, medium effect ~0.64, and large effect ~0.71.

Linear relationships between training years and posturography results at PRE were tested by computing Spearman rank correlation coefficients. Similarly, Spearman rank correlation analysis between the number of training years and learning rates during DBT (%-wise improvement from training sessions 1–9) was performed.

The statistical threshold for all analyses was set at *p*<0.05 and was appropriately Bonferroni adjusted to correct for multiple *post-hoc* comparisons.

## Results

Football group and GG did not differ in demographic and anthropometric variables, i.e., age, height, weight, and handedness (all *p*>0.05). However, groups differed in the number of training years (FG: 5.1±2.1 vs. GG: 6.0±0.8, *W*=122.5, *p*=0.022, *r*=0.419).

### Initial Assessment of Postural Stability: Group Comparison Football vs. Gymnastics

Initial performances (COP path length) in both two-legged stance conditions (TLS-EO and TLS-EC) did not differ between FG and GG (TLS-EO: 510.5mm vs. 456.0mm, *W*=248.0, *p*=0.328, *r*=0.181; TLS-EC: 576.0mm vs. 531.0mm, *W*=248.5, *p*=0.322, *r*=0.183).

**Figure 2:**
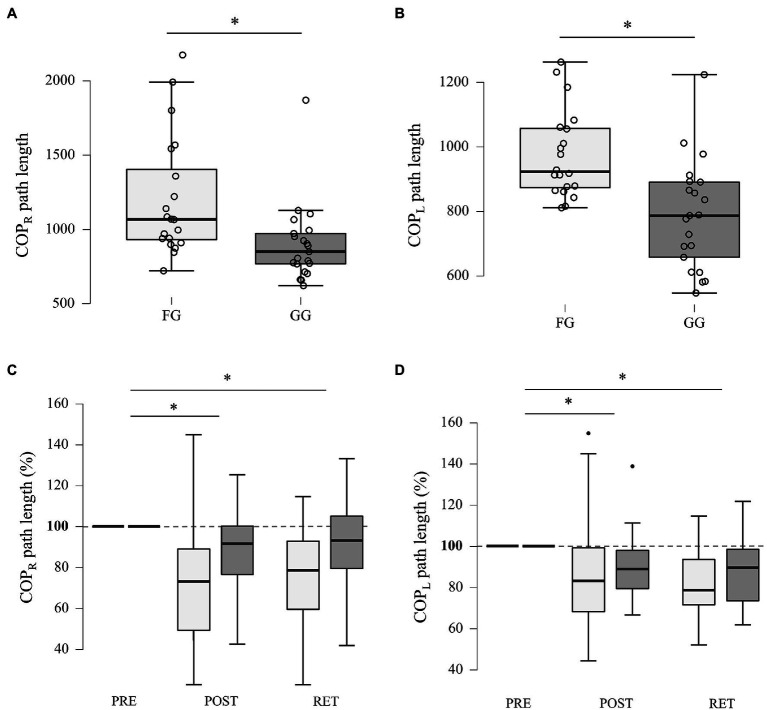
Posturography results. **(A)** Group comparison of the initial performance during one-legged stance postural stability with the right leg (OLS-R). **(B)** Group comparison of the initial performance during one-legged stance postural stability with the left leg (OLS-L). **(C)** Changes in postural stability during OLS-R induced by dynamic balance training. Values obtained at POST and RET were normalized to PRE values. **(D)** Changes in postural stability during OLS-L induced by dynamic balance training. Values obtained at POST and RET were normalized to PRE values. ^*^ indicates significant comparison.

However, GG showed significantly less COP path lengths during both one-legged stance conditions (OLS-R and OLS-L; Figure 2). During OLS-R, GG had a path length of 851.0mm compared to 1,068.5mm observed in the FG (*W*=326.5, *p*=0.002, *r*=0.555). During OLS-L, 787.0mm in the GG were observed, compared to 923.5mm in the FG (*W*=345.0, *p*<0.001, *r*=0.643).

### Dynamic Balance Training

**Figure 3:**
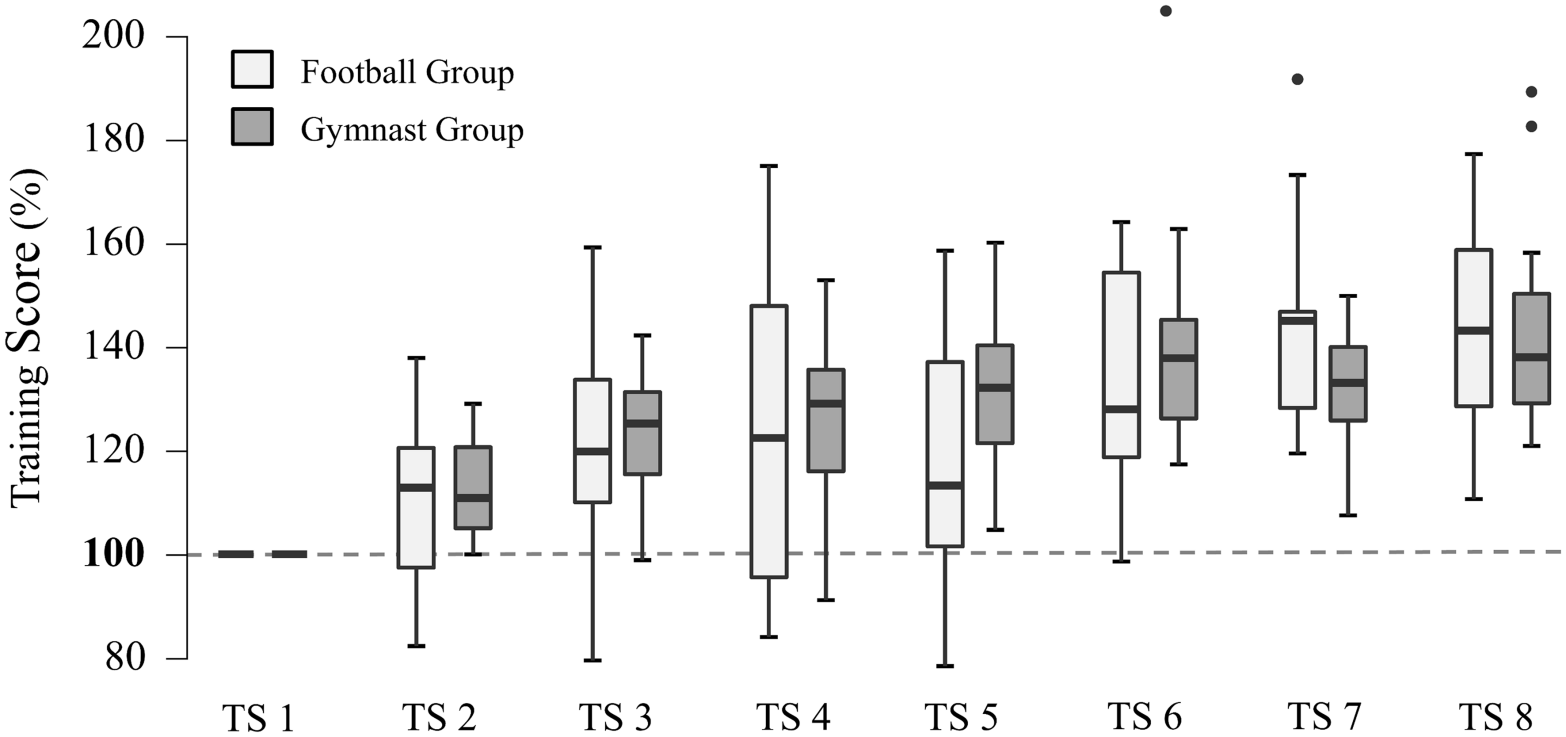
Dynamic balance training results. Significant improvements in training scores of dynamic balance training using the challenge disk over eight training sessions (TS). All values were normalized to scores obtained during TS 1. For detailed pairwise *post-hoc* statistics please see Table 1.

Dynamic balance training over 4weeks induced a highly significant TIME effect (Figure 3), more precisely an increase in training scores (*F*_5.161, ∞_=92.141, *p*<0.001), but no significant GROUP effect was observed (*F*_1.000, ∞_=1.188, *p*=0.278). Additionally, a significant interaction TIME×GROUP was found (*F*_5.161, ∞_=2.251, *p*=0.045). However, interaction *post-hoc* tests failed to reach significance. For pairwise *post-hoc* comparisons statistics of the Factor TIME, please see Table 1.

**Table 1:** Pairwise *post-hoc* comparisons of the within-subject (sub-plot) factor TIME (training sessions=TS) on compound training scores (summarized time spent in the target zone for all nine protocols) during dynamic balance training.

Pairwise comparison (TIME)	Value of *p*	*F* ^a^	*A* ^b^
TS 1–TS 2	<0.001^*^	61.68	0.85
TS 2–TS 3	<0.001^*^	12.17	0.70
TS 3–TS 4	0.669	0.18	0.52
TS 4–TS 5	0.590	0.29	0.53
TS 5–TS 6	<0.001^*^	12.17	0.64
TS 6–TS 7	0.480	0.50	0.53
TS 7–TS 8	0.024	5.10	0.59

* Significant comparison, significance level Bonferroni-adjusted for seven *post-hoc* comparisons to *α*=0.00625.

a ANOVA-type statistic.

b Vargha–Delaney effect size of stochastic superiority.

### Effects of Dynamic Balance Training on Postural Stability

ANOVA-type statistics indicated no significant effect of the factor GROUP (*F*_1.000, ∞_=0.008, *p*=0.926) or TIME (*F*_1.729, ∞_=9.102, *p*=0.058) on posturography results during TLS-EO. Furthermore, no interaction effect GROUP×TIME was found (*F*_1.729, ∞_=0.010, *p*=0.982).

Regarding TLS-EC, no effect was found for the factor GROUP (*F*_1.000, ∞_=0.920, *p*=0.337), TIME (*F*_1.997, ∞_=2.176, *p*=0.114), and the interaction effect GROUP×TIME (*F*_1.997, ∞_=1.123, *p*=0.325), indicating the absence of any DBT-induced changes of two-legged stance postural stability.

For OLS-R, ATS indicated a significant effect of TIME on postural stability (*F*_1.403, ∞_=35.542, *p*<0.001). Pairwise *post-hoc* comparisons for the factor TIME showed a significant improvement in postural stability for PRE vs. POST (*F*_1.000, ∞_=48.08, *p*<0.001, *A*=0.83) and for PRE vs. RET (*F*_1.000, ∞_=25.30, *p*<0.001, *A*=0.76). However, no significant effect of GROUP (*F*_1.000, ∞_=2.938, *p*=0.086) or interaction effect GROUP×TIME (*F*_1.403, ∞_=2.592, *p*=0.094) was found.

The same results were found for OLS-L, with a significant TIME effect (*F*_1.658, ∞_=40.401, *p*<0.001), no significant effect for GROUP (*F*_1.000, ∞_=1.363, *p*=0.243) and a non-significant interaction GROUP×TIME (*F*_1.659, ∞_=1.494, *p*=0.226), indicating that DBT improved one-legged stance postural stability of both legs in both groups. Pairwise *post-hoc* comparisons for the factor TIME again showed a significant improvement in postural stability for PRE vs. POST (*F*_1.000, ∞_=33.05, *p*<0.001, *A*=0.79) and for PRE vs. RET (*F*_1.000, ∞_=64.99, *p*<0.001, *A*=0.85).

### Relationship Between Training Age and Balance Performance

The number of training years significantly correlated negatively with all posturography variables obtained at PRE (TLS-EO: *r_s_*=−0.382, *p*=0.014, 95% CI [−0.617, −0.084]; TLS-EC: *r_s_*=−0.412, *p*=0.007, 95% CI [−0.639, −0.120]; OLS-R: *r_s_*=−0.583, *p*<0.001, 95% CI [−0.759, −0.343]; OLS-L: *r_s_*=−0.495, *p*=0.001, 95% CI [−0.696, −0.221]). This means that the longer participants trained in their respective sport, the shorter their COP path length during the initial assessment of postural stability.

Dynamic balance training learning rates (%-wise improvement from training session 1 to training session 8) and the number of training years were not significantly correlated (*r*_s_=0.071, *p*=0.657, 95% CI [−0.242, 0.371]).

## Discussion

In the present study, we aimed to investigate the effects of a 4-week DBT on static balance performance in school-aged gymnasts and football players. Our findings demonstrate a significant effect of DBT on static balance performance in GG and FG. Initially, static balance performance was enhanced for OLS in GG compared to FG. DBT led to an improvement in dynamic balance performance in both groups. Regarding possible effects of DBT on static balance, one-legged stance performance was significantly improved in both groups, while two-legged stance showed no differences between PRE and POST. In addition, retention rates were high in both groups for one-legged stance (left and right) but not significant for two-legged stance. All findings and their implications are discussed below.

Initial OLS performance was greater in GG compared to FG. This was to be expected, as previous studies showed greater overall balance ability in gymnasts compared to football players (Hrysomallis, 2011). It can be argued that this finding relates to the differences in balance demands between gymnasts and football players (Bressel et al., 2007). However, GG had significantly more training years compared to FG. Therefore, we correlated the years of training and the initial OLS performance and found a significant negative correlation. That is, the longer the athletes trained, the better their initial performance was, for both OLS-L and OLS-R. Consequently, since gymnasts often practice both stable postures and static balance (Ashton-Miller et al., 2001) and had more training experience in our study, it is conceivable that a combination of superior initial ability and greater experience may account for the better initial performance of GG in OLS.

Dynamic balance performance increased in both groups throughout the intervention. We expected that both groups would significantly improve dynamic balance performance because both gymnastics and football contain many elements related to dynamic balance, and dynamic balance development is integrated into common training regimens in both sports (Bressel et al., 2007). Although it can be assumed that athletes generally have higher balance abilities than non-athletes (Hrysomallis, 2011), a recent meta-analysis has shown that improvements in balance abilities in response to organized balance training are irrespective of training status (Gebel et al., 2018). However, learning rates did not differ between groups. We anticipated differences in learning rates as previous studies showed gymnasts to have better balance ability compared to football players (Hrysomallis, 2011). As learning rates relate to the initial performance abilities (Dayan and Cohen, 2011), we therefore assumed greater potential for improvement and consequently higher learning rates for FG. The absence of such effects could relate to statistical power since we found a significant TIME×GROUP interaction whereas *post-hoc* tests failed to reach significance. Compared to other studies on balance ability, however, our sample size is adequate (Hrysomallis, 2011). In addition, several studies emphasize the importance of task specificity in improving sport-specific balance performance (Giboin et al., 2015; Freyler et al., 2016; Kümmel et al., 2016). In this study, we used a general DBT, meaning that the balance tasks included were nonspecific for both gymnasts and football players. For this reason, it can be assumed that learning rates did not differ due to the lack of task specificity, as none of our athlete groups were favored by the task. Nevertheless, it is recommended that future studies use larger samples to address this potential issue. Lastly, to investigate a potential influence of the number of training years on DBT learning rates, we correlated both factors. However, our results did not show a statistically significant relationship. This might be explained since all participants were task naïve concerning the DBT protocols. Therefore, the number of training years within their specific sport did not significantly affect learning rates in the unrelated DBT.

Although learning rates did not differ between groups, DBT lead to an improvement in static balance in both GG and FG. Specifically, GG and FG significantly improved in OLS-L and OSL-R performance while no improvements were observed for TLS-EO or TLS-EC. These results are in line with previous studies that demonstrated significant improvements in static balance following dynamic balance interventions (Battaglia et al., 2010; Bellafiore et al., 2011). For the first time, our results extend these findings to young athletes of different sports. Notably, previous studies imply limited transfer effects between different components of balance ability (Horak et al., 2009; Giboin et al., 2015). Many authors associate this limit of transfer with specific adaptations of the motor system following different balance tasks (Giboin et al., 2015). However, it has been shown that after balance training, improvement in balance ability is related to improvement in other components of athletic performance, such as strength (Gusi et al., 2012), rate of force development (Gruber and Gollhofer, 2004), and proprioception (Emilio et al., 2014). It is therefore tempting to speculate that the improvements in static balance observed in our study following DBT are related to improvements in other components of physical performance. This is supported by the fact that dynamic balance training has been shown to improve both strength and sensory processing (Hamed et al., 2018). Notably, our findings not only show a relationship between static and dynamic balance but also highlight subtle distinctions. In particular, it is interesting to note that only OLS was positively affected by DBT. OLS comprises more dynamic balance components compared to TLS due to the higher number of degrees of freedom (García-Massó et al., 2016). For this reason, the requirement for dynamic balance regulation is higher in OLS, which potentially explains the improved performance of both groups in OLS. Further, both sports incorporate a considerable amount of unipedal movements, e.g., passing, receiving, and shooting in football (Orchard, 2002), as well as balancing, leaping, and tumbling in gymnastics (Hay, 1978). Accordingly, both gymnasts and football players may have an increased ability to manage unipedal stabilization.

Lastly, retention rates for OLS were high in both groups with no significant difference between groups, whereas no effects on retention rates were found for TLS. High retention rates were expected as previous research demonstrated enhanced retention rates in children for a number of balance tasks (Rodríguez-Negro et al., 2020). Children seem to be particularly adept at retaining a learned skill, and oftentimes skill acquisition leads to permanent changes in their ability to perform the learned skill (Rodríguez-Negro et al., 2020).

### Limitations

In the following, some limitations of this study are addressed in order to delimit the scope of the results as well as to ensure guidelines for follow-up studies. Main limitations of studies on balance ability like the present study are in the area of intervention design. To date, there is no gold standard for optimizing the development of static or dynamic balance ability (Brachman et al., 2017). Variable elements include training intervention, duration of a single session, and total duration of the intervention. Due to the novelty of our study, we kept these parameters as simple as possible, following current literature, in order to facilitate conclusions. Nevertheless, it must be noted that the results presented here are by no means generalizable and should be supported by additional studies investigating the effect of divergently chosen interventions on transferability between static and dynamic balance ability. With this in mind, young female athletes also need to be studied. Due to the known differences in static balance between girls and boys (Humphriss et al., 2011), we decided in this study to first test a cohort of boys in order to examine girls in a follow-up study and compare the results.

## Conclusion

In the present study, we demonstrate for the first time a positive effect of 4weeks of DBT on one-legged static balance performance in young gymnasts and football players. Our results shed light on the interplay between dynamic and static balance ability in both sports, as we show improvements in static balance following DBT. These results may aid in optimizing training regimens related to balance performance. Future studies should consider extending our paradigm to other sports to consolidate and generalize our findings.

## Data Availability Statement

The datasets presented in this article are not readily available because of data protection policies in the ethics agreement. Requests to access the datasets should be directed to tom.maudrich@uni-leipzig.de.

## Ethics Statement

The studies involving human participants were reviewed and approved by the Ethics Committee at the Medical Faculty of the University of Leipzig, Stephanstraße 9A.1, 04103 Leipzig. Written informed consent to participate in this study was provided by the participants’ legal guardian/next of kin.

## Author Contributions

SK and PR designed the study and provided critical revision. SK acquired the data. RK and TM analyzed the data. RK, TM, and JZ wrote the manuscript. All authors interpreted the data, contributed to the manuscript, reviewed the manuscript, approved the content of the final version, and agreed to be accountable for all aspects of the work. All persons designated as authors qualify for authorship, and all those who qualify for authorship are listed. All authors contributed to the article and approved the submitted version.

## Conflict of Interest

The authors declare that the research was conducted in the absence of any commercial or financial relationships that could be construed as a potential conflict of interest.

## Publisher’s Note

All claims expressed in this article are solely those of the authors and do not necessarily represent those of their affiliated organizations, or those of the publisher, the editors and the reviewers. Any product that may be evaluated in this article, or claim that may be made by its manufacturer, is not guaranteed or endorsed by the publisher.

## References

[ref1] Akbas K. Mummolo C. (2021). A computational framework towards the tele-rehabilitation of balance control skills. Front. Robot. AI 8:648485. doi: 10.3389/frobt.2021.648485, PMID: 34179106 PMC8220374

[ref2] Ashton-Miller J. A. Wojtys E. M. Huston L. J. Fry-Welch D. (2001). Can proprioception really be improved by exercises? Knee Surg. Sports Traumatol. Arthrosc. 9:128. doi: 10.1007/s001670100208, PMID: 11420785

[ref3] Bathke A. C. Schabenberger O. Tobias R. D. Madden L. V. (2009). Greenhouse–Geisser adjustment and the ANOVA-type statistic: cousins or twins? Am. Stat. 63, 239–246. doi: 10.1198/tast.2009.08187

[ref4] Battaglia G. Bellafiore M. Bianco A. Paoli A. Palma A. (2010). Effects of a dynamic balance training protocol on podalic support in older women. Pilot study. Aging Clin. Exp. Res. 22, 406–411. doi: 10.1007/BF03337736, PMID: 20009497

[ref5] Bayer U. Likar R. Pinter G. Stettner H. Demschar S. Trummer B.. (2017). Intermittent hypoxic–hyperoxic training on cognitive performance in geriatric patients. Alzheimers Dement. 3, 114–122. doi: 10.1016/j.trci.2017.01.002, PMID: 29067323 PMC5651371

[ref6] Bellafiore M. Battaglia G. Bianco A. Paoli A. Farina F. Palma A. (2011). Improved postural control after dynamic balance training in older overweight women. Aging Clin. Exp. Res. 23, 378–385. doi: 10.1007/BF03337762, PMID: 21084833

[ref7] Brachman A. Kamieniarz A. Michalska J. Pawlowski M. Slomka K. J. Juras G. (2017). Balance training programs in athletes—a systematic review. J. Hum. Kinet. 58, 45–64. doi: 10.1515/hukin-2017-0088, PMID: 28828077 PMC5548154

[ref8] Bressel E. Yonker J. C. Kras J. Heath E. M. (2007). Comparison of static and dynamic balance in female collegiate soccer, basketball, and gymnastics athletes. J. Athl. Train. 42, 42–46. PMID: 17597942 PMC1896078

[ref9] Brunner E. Domhof S. Langer F. (2002). Nonparametric Analysis of Longitudinal Data in Factorial Experiments Hoboken, New Jersey, USA: Wiley-Interscience.

[ref10] Butz S. M. Sweeney J. K. Roberts P. L. Rauh M. J. (2015). Relationships among age, gender, anthropometric characteristics, and dynamic balance in children 5 to 12 years old. Pediatr. Phys. Ther. 27, 126–133. doi: 10.1097/PEP.0000000000000128, PMID: 25695196

[ref11] Cankaya S. Gokmen B. Tasmektepligil M. Y. Con M. (2015). Special balance developer training applications on young males’ static and dynamic balance performance. Anthropologist 19, 31–39. doi: 10.1080/09720073.2015.11891636

[ref12] Chang W.-D. Chang W.-Y. Lee C.-L. Feng C.-Y. (2013). Validity and reliability of wii fit balance board for the assessment of balance of healthy young adults and the elderly. J. Phys. Ther. Sci. 25, 1251–1253. doi: 10.1589/jpts.25.1251, PMID: 24259769 PMC3820194

[ref13] Clark R. A. Bryant A. L. Pua Y. McCrory P. Bennell K. Hunt M. (2010). Validity and reliability of the Nintendo Wii balance board for assessment of standing balance. Gait Posture 31, 307–310. doi: 10.1016/j.gaitpost.2009.11.012, PMID: 20005112

[ref14] Dayan E. Cohen L. G. (2011). Neuroplasticity subserving motor skill learning. Neuron 72, 443–454. doi: 10.1016/j.neuron.2011.10.008, PMID: 22078504 PMC3217208

[ref15] DiStefano L. J. Clark M. A. Padua D. A. (2009). Evidence supporting balance training in healthy individuals: a systemic review. J. Strength Cond. Res. 23, 2718–2731. doi: 10.1519/JSC.0b013e3181c1f7c5, PMID: 19910803

[ref16] Dobrijević S. Moskovljević L. Dabović M. (2016). The influence of proprioceptive training on young rhythmic gymnasts balance. Facta Univ. Ser. Phys. Educ. Sport 14, 247–255.

[ref17] Donath L. Roth R. Rueegge A. Groppa M. Zahner L. Faude O. (2013). Effects of slackline training on balance, jump performance & muscle activity in young children. Int. J. Sports Med. 34, 1093–1098. doi: 10.1055/s-0033-1337949, PMID: 23700328

[ref18] Emilio E. J. Hita-Contreras F. Jimenez-Lara P. M. Latorre-Roman P. Martinez-Amat A. (2014). The association of flexibility, balance, and lumbar strength with balance ability: risk of falls in older adults. J. Sports Sci. Med. 13, 349–357. PMID: 24790489 PMC3990889

[ref19] Faigenbaum A. D. Bagley J. Boise S. Farrell A. Bates N. Myer G. D. (2015). Dynamic balance in children: performance comparison between two testing devices. Athlet. Train. Sports Health Care 7, 160–164. doi: 10.3928/19425864-20150707-06

[ref20] Faul F. Erdfelder E. Buchner A. Lang A.-G. (2009). Statistical power analyses using G* power 3.1: tests for correlation and regression analyses. Behav. Res. Methods 41, 1149–1160. doi: 10.3758/BRM.41.4.1149, PMID: 19897823

[ref21] Freyler K. Krause A. Gollhofer A. Ritzmann R. (2016). Specific stimuli induce specific adaptations: sensorimotor training vs. reactive balance training. PLoS One 11:e0167557. doi: 10.1371/journal.pone.0167557, PMID: 27911944 PMC5135127

[ref22] García-Massó X. Pellicer-Chenoll M. Gonzalez L. Toca-Herrera J. (2016). The difficulty of the postural control task affects multi-muscle control during quiet standing. Exp. Brain Res. 234, 1977–1986. doi: 10.1007/s00221-016-4602-z, PMID: 26942928 PMC4893067

[ref23] Gebel A. Lesinski M. Behm D. G. Granacher U. (2018). Effects and dose–response relationship of balance training on balance performance in youth: a systematic review and meta-analysis. Sports Med. 48, 2067–2089. doi: 10.1007/s40279-018-0926-0, PMID: 29736728

[ref24] Giboin L.-S. Gruber M. Kramer A. (2015). Task-specificity of balance training. Hum. Mov. Sci. 44, 22–31. doi: 10.1016/j.humov.2015.08.012, PMID: 26298214

[ref25] Granacher U. Muehlbauer T. Maestrini L. Zahner L. Gollhofer A. (2011). Can balance training promote balance and strength in prepubertal children? J. Strength Cond. Res. 25, 1759–1766. doi: 10.1519/JSC.0b013e3181da7886, PMID: 21386732

[ref26] Gruber M. Gollhofer A. (2004). Impact of sensorimotor training on the rate of force development and neural activation. Eur. J. Appl. Physiol. 92, 98–105. doi: 10.1007/s00421-004-1080-y, PMID: 15024669

[ref27] Gusi N. Carmelo Adsuar J. Corzo H. Del Pozo-Cruz B. Olivares P. R. Parraca J. A. (2012). Balance training reduces fear of falling and improves dynamic balance and isometric strength in institutionalised older people: a randomised trial. J. Phys. 58, 97–104. doi: 10.1016/S1836-9553(12)70089-9, PMID: 22613239

[ref28] Hamed A. Bohm S. Mersmann F. Arampatzis A. (2018). Exercises of dynamic stability under unstable conditions increase muscle strength and balance ability in the elderly. Scand. J. Med. Sci. Sports 28, 961–971. doi: 10.1111/sms.13019, PMID: 29154407

[ref29] Hay J. (1978). The Biomechanics of Sports Techniques Hoboken, New Jersey, USA: Prentice-Hall.

[ref30] Heleno L. R. da Silva R. A. Shigaki L. Araújo C. G. A. Candido C. R. C. Okazaki V. H. A.. (2016). Five-week sensory motor training program improves functional performance and postural control in young male soccer players–A blind randomized clinical trial. Phys. Ther. Sport 22, 74–80. doi: 10.1016/j.ptsp.2016.05.004, PMID: 27620862

[ref31] Horak F. B. (1987). Clinical measurement of postural control in adults. Phys. Ther. 67, 1881–1885. doi: 10.1093/ptj/67.12.1881, PMID: 3685116

[ref32] Horak F. B. Wrisley D. M. Frank J. (2009). The balance evaluation systems test (BESTest) to differentiate balance deficits. Phys. Ther. 89, 484–498. doi: 10.2522/ptj.20080071, PMID: 19329772 PMC2676433

[ref33] Hrysomallis C. (2011). Balance ability and athletic performance. Sports Med. 41, 221–232. doi: 10.2165/11538560-000000000-00000, PMID: 21395364

[ref34] Humphriss R. Hall A. May M. Macleod J. (2011). Balance ability of 7 and 10 year old children in the population: results from a large UK birth cohort study. Int. J. Pediatr. Otorhinolaryngol. 75, 106–113. doi: 10.1016/j.ijporl.2010.10.019, PMID: 21074865

[ref35] Kalisch T. Kattenstroth J.-C. Noth S. Tegenthoff M. Dinse H. R. (2011). Rapid assessment of age-related differences in standing balance. J. Aging Res. 2011:160490. doi: 10.4061/2011/160490, PMID: 21629742 PMC3100560

[ref36] Kim K. Cha Y. J. Fell D. W. (2011). The effect of contralateral training: influence of unilateral isokinetic exercise on one-legged standing balance of the contralateral lower extremity in adults. Gait Posture 34, 103–106. doi: 10.1016/j.gaitpost.2011.03.022, PMID: 21536441

[ref37] Kümmel J. Kramer A. Giboin L.-S. Gruber M. (2016). Specificity of balance training in healthy individuals: a systematic review and meta-analysis. Sports Med. 46, 1261–1271. doi: 10.1007/s40279-016-0515-z, PMID: 26993132

[ref38] Largo R. H. Caflisch J. A. Hug F. Muggli K. Molnar A. A. Molinari L.. (2001). Neuromotor development from 5 to 18 years. Part 1: timed performance. Dev. Med. Child Neurol. 43, 436–443. doi: 10.1017/S0012162201000810, PMID: 11463173

[ref39] Le Clair K. Riach C. (1996). Postural stability measures: what to measure and for how long. Clin. Biomech. 11, 176–178. doi: 10.1016/0268-0033(95)00027-5, PMID: 11415618

[ref40] Noguchi K. Gel Y. R. Brunner E. Konietschke F. (2012). nparLD: an R software package for the nonparametric analysis of longitudinal data in factorial experiments. J. Stat. Softw. 50, 1–23. doi: 10.18637/jss.v050.i1225317082

[ref41] Oldfield R. C. (1971). The assessment and analysis of handedness: the Edinburgh inventory. Neuropsychologia 9, 97–113. doi: 10.1016/0028-3932(71)90067-4, PMID: 5146491

[ref42] Orchard J. (2002). Is there a relationship between ground and climatic conditions and injuries in football? Sports Med. 32, 419–432. doi: 10.2165/00007256-200232070-00002, PMID: 12015804

[ref43] Paillard T. (2017). Plasticity of the postural function to sport and/or motor experience. Neurosci. Biobehav. Rev. 72, 129–152. doi: 10.1016/j.neubiorev.2016.11.015, PMID: 27894829

[ref44] Pau M. Loi A. Pezzotta M. C. (2012). Does sensorimotor training improve the static balance of young volleyball players? Sports Biomech. 11, 97–107. doi: 10.1080/14763141.2011.637126, PMID: 22518948

[ref45] Rodríguez-Negro J. Pesola J. A. Yanci J. (2020). Effects and retention of different physical exercise programs on children’s cognitive and motor development. J. Educ. Res. 113, 431–437. doi: 10.1080/00220671.2020.1854159

[ref46] Sannicandro I. Cofano G. Rosa R. A. Piccinno A. (2014). Balance training exercises decrease lower-limb strength asymmetry in young tennis players. J. Sports Sci. Med. 13, 397–402. PMID: 24790496 PMC3990896

[ref47] Seidel O. Carius D. Kenville R. Ragert P. (2017). Motor learning in a complex balance task and associated neuroplasticity: A comparison between endurance athletes and non-athletes. J. Neurophysiol. 118, 1849–1860. doi: 10.1152/jn.00419.2017, PMID: 28659467 PMC5599667

[ref48] Shumway-Cook A. Woollacott M. H. (1985). The growth of stability: postural control from a developmental perspective. J. Mot. Behav. 17, 131–147. doi: 10.1080/00222895.1985.10735341, PMID: 15140688

[ref49] Team R. (2020). RStudio: Integrated Development Environment for R (RStudio, PBC).

[ref50] Vargha A. Delaney H. D. (2000). A critique and improvement of the CL common language effect size statistics of McGraw and Wong. J. Educ. Behav. Stat. 25, 101–132. doi: 10.3102/10769986025002101

[ref51] Wälchli M. Ruffieux J. Mouthon A. Keller M. Taube W. (2018). Is young age a limiting factor when training balance? Effects of child-oriented balance training in children and adolescents. Pediatr. Exerc. Sci. 30, 176–184. doi: 10.1123/pes.2017-0061, PMID: 28605259

